# Better therapy for combat injury

**DOI:** 10.1186/s40779-019-0214-9

**Published:** 2019-07-25

**Authors:** Yong-ming Yao, Hui Zhang

**Affiliations:** Trauma Research Center, Fourth Medical Center of the Chinese PLA General Hospital, Fucheng Road 51, Haidian District, Beijing, 100048 People’s Republic of China

**Keywords:** Combat injury, Sepsis, Multiple organ dysfunction syndrome, Estrogen, Immune dysfunction, Mitochondrial function, Interleukin-25

## Abstract

In modern warfare, therapy for combat injury is a critical issue to improve personnel survival and battle effectiveness. Be limited to the severe circumstance in the distant battlefield, quick and effective treatment cannot be supplied that leads infections, sepsis, multiple organ dysfunction syndrome (MODS) and high mortality. To get a better therapy for combat injury, we summarized several reports that associated with the mechanisms of sepsis and MODS, those published on MMR recently. Chaudry and colleagues reported gender difference in the outcomes of trauma, shock and sepsis. The advantageous outcome in female is due to their hormone milieu. Their accumulating reports indicated estrogen as a beneficial factor for multiple system and organs, including the central nervous system, the cardiopulmonary system, the liver, the kidneys, the immune system, and leads to better survival from sepsis. Thompson et al. reviewed the underlying mechanisms in trauma induced sepsis, which can be concluded as an imbalance of immune response triggered by damage-associated molecular patterns (DAMPs) and other immune modifying agents. They also emphasize immunomodulation as a better therapeutic strategy that might be a potential benefit in regulating the host immune response. Fan et al. have revealed a crucial mechanism underlying lung epithelial and macrophage crosstalk, which involves IL-25 as a mediator. After the injury, lung epithelial secreted IL-25 promotes TNF-α production in macrophage leading to acute lung injury (ALI). In addition to a mountain of cytokines, mitochondrial dysfunction in immune cell is another critical risk factor for immune dysfunction during sepsis. Both morphology and function alterations in mitochondria are closely associated with inadequate ATP production, insufficient metabolism process and overloaded  ROS production, which lead harm to immune cells and other tissues by triggering oxidative stress. All the above reports discussed mechanisms of sepsis induction after trauma and provided evidence to improve better therapy strategies targeting diverse risk factors.

Trauma and hemorrhage are common combat injury, which might lead to financial burdens and fatal outcomes. In remote military situations, delayed transportation is a major challenge for the management of trauma and hemorrhagic shock, which require large amounts of fluid and blood product, allowing permissive hypotension before arrival at the final medical facility. Often, adequate fluid support cannot be supplied in time due to the severe circumstances on the battlefield. Even if successful resuscitation and simple operations are possible, post injury infection, sepsis and multiple organ dysfunction syndrome (MODS) contribute to morbidity in those who survive the initial trauma. Therefore, timely and sufficient fluid support or better replacement therapy is required for trauma patients in modern warfare and in distant accidents. Furthermore, the prevention of subsequent septic complications is a critical issue for improving outcomes.

Recently, Chaudry and colleagues [1, 2] reported a beneficial effect of estrogen on reserving permissive hypotension via the inhibition of cardiac apoptosis after trauma and hemorrhage. Although the authors’ findings are based on animal experiments, the underlying mechanism has been well illustrated for decades by their groups. In a recent review, the authors deeply analyzed the gender differences in trauma, hemorrhagic shock, and sepsis [3]. For decades, the authors have noticed better outcomes in female patients. However, some contradictory clinical trials have demonstrated that females are at risk for mortality in cases of spontaneous bacterial peritonitis, which ignores the hormonal status according to age. As a result, the authors realized that hormones but not gender might be the key factor for outcomes. Estrogen plays a protective role in multiple systems, including the central nervous system, cardiovascular system, respiratory system, renal and immune systems, by reducing the production of pro-inflammatory cytokines, enhancing the expression of heat shock proteins (HSPs) and heme oxygenase-1 (HO-1), and protecting against cell apoptosis. Accordingly, treatment with estrogen might be possible for patients with trauma and hemorrhage, especially in combat casualties, and it improves survival by maintaining the function of multiple system. Another important implication is that this approach may be implemented at the scene of an accident to stabilize injured patients with hemorrhage, even in the absence of resuscitation, thereby prolonging the permissive hypotension period.

For future development in therapeutic strategies for the prevention of sepsis and MODS, it is indispensable to understand the underlying pathophysiology mechanism. In the review article, Thompson et al. [4] indicated immune dysfunction as a key risk factor for the late onset of infection, sepsis, and MODS after trauma. In combat casualties, delaying suitable treatment may result in prolonged immune dysfunction with subsequent late complications, such as wound infection, delayed wound healing, sepsis, and MODS. With regard to the mechanism underlying immune depression, damage-associated molecular patterns (DAMPs) and large amounts of cytokines are initial factors that promote the disturbance of the immune response. Additionally, a lower expression of human histocompatibility leukocyte antigen (HLA)-DR on monocytes and lymphocyte dysfunction appears to be involved in the development of immune paralysis. Based on these theories, immunomodulation is revealed to be a better therapeutic strategy for septic complications in the setting of acute insults. Emerging evidence from clinical trials has shown that some cytokines might be of potential benefit in regulating the host immune response, including granulocyte colony-stimulating factor (CSF) /granulocyte-macrophage CSF, interferon (IFN)-γ, interleukin (IL)-7, IL-15, thymosin α1, etc.

Accumulated reports discover unidentified cytokines or reveal their novel roles in the immune response. The majority of the research is focused on the direct effects on immune cell differentiation or activation. Fan and his group [5] have recognized IL-25 as a crucial mediator in the inflammatory response in acute lung injury (ALI), which is a major component of MODS following trauma and infection. It is well known that exosomes are cell-derived, secreted vesicles with bi-lipid membranous structures that contain RNA, proteins, and lipids. The researchers previously documented a crosstalk pathway between macrophages and neutrophils via exosomes in hemorrhagic shock [6]. Recently, they found that IL-25 secreted by lung epithelial cells (LEPCs) down-regulated Rab27a and Rab27b expression in macrophages, thereby inhibiting exosome-mediated tumor necrosis factor (TNF)-α secretion from macrophages. Therefore, these findings provide new targets for the treatment of ALI by modulating IL-25 signaling and exosomes.

In addition to the abovementioned mechanism(s), host immune function can be regulated by multiple factors that still need further exploration. Of note, mitochondrial function is critical for cell metabolism and energy production, and it appears to be associated with redox signaling, calcium flux, and apoptosis [7]. In trauma, hemorrhagic shock, and sepsis, hypoxia induces immune cell apoptosis and dysfunction, which greatly involves alterations in mitochondrial stability and function. Moreover, the uncompleted metabolism of oxygen and nutrients promotes oxidative stress that further harms mitochondria per se and regulates the immune response. To date, several methods of monitoring mitochondrial function have been developed and implicated in animal experiments, and they might be further improved for clinical application. Accordingly, novel therapeutic strategies through the improvement of mitochondrial function are beneficial for protecting host immunity and organ function, such as mitochondrial membrane channel blockers, electric transport chain (ETC) enzymes, antioxidants, and biogenesis promotion reagents. The most attractive reagents among them are mitochondria-targeted coenzymes that have already been proven to be safe and are beneficial for various diseases.

In modern warfare, the combat injury pattern is complicated and always combines several injuries, including extensive burns, multiple trauma, bone fracture, hemorrhage, infection, sepsis, and organ damage, each of which can activate the immune response. In a severe and combined injury, immune response disturbance occurs, and finally, immunosuppression leads to sepsis and MODS. In the wake of revealing a possible mechanism concerning immune dissonance, we have understood that immune modulation is vital and indispensable in the treatment of sepsis and MODS, which should not be limited in exploring cytokines and molecules directly regulating immune cells. Other factors involved in immunomodulation must also be considered; for instance, cellular biological processes are markedly changed in acute insults and contribute to the responsiveness of immune cells, including metabolism, apoptosis, necroptosis, autophagy, and intercell communications. Taken together, the reviews and research in the current thematic series shed light on the exploration of novel interventional strategies for better therapy for combat injury. Although there remains a gap between the bench and the bedside or battlefield, it is our belief that a comprehensive consideration must lead to the significant development of clinical therapies in the future.

## Data Availability

Not applicable.

## Abbreviations

ALI: Acute lung injury
CSF: Colony-stimulating factor
DAMPs: Damage-associated molecular patterns
ETC: Electric transport chain
HLA: Histocompatibility leukocyte antigen
HO-1: Heme oxygenase-1
HSPs: Heat shock proteins
IFN: Interferon
IL: Interleukin
LEPCs: Lung epithelial cells
MODS: Multiple organ dysfunction syndrome
TNF: Tumor necrosis factor
